# Basal Sodium-Dependent Vitamin C Transporter 2 polarization in choroid plexus explant cells in normal or scorbutic conditions

**DOI:** 10.1038/s41598-019-50772-2

**Published:** 2019-10-08

**Authors:** Viviana Ulloa, Natalia Saldivia, Luciano Ferrada, Katterine Salazar, Fernando Martínez, Carmen Silva-Alvarez, Rocio Magdalena, María José Oviedo, Hernán Montecinos, Pablo Torres-Vergara, Manuel Cifuentes, Francisco Nualart

**Affiliations:** 1 Laboratory of Neurobiology and Stem Cells NeuroCellT, Department of Cellular Biology, Faculty of Biological Sciences, University of Concepcion, Concepcion, Chile; 2 Center for Advanced Microscopy CMA BIO BIO, University of Concepcion, Concepcion, Chile; 3 Department of Cellular Biology, Faculty of Biological Sciences, University of Concepcion, Concepcion, Chile; 4 Department of Cell Biology, Genetics and Physiology, University of Malaga, IBIMA, BIONAND, Andalusian Center for Nanomedicine and Biotechnology and Networking Research Center on Bioengineering, Biomaterials and Nanomedicine, (CIBER-BBN), Malaga, Spain

**Keywords:** Blood-brain barrier, Blood-brain barrier, Cellular neuroscience, Cellular neuroscience

## Abstract

Vitamin C is incorporated into the cerebrospinal fluid (CSF) through choroid plexus cells. While the transfer of vitamin C from the blood to the brain has been studied functionally, the vitamin C transporter, SVCT2, has not been detected in the basolateral membrane of choroid plexus cells. Furthermore, it is unknown how its expression is induced in the developing brain and modulated in scurvy conditions. We concluded that SVCT2 is intensely expressed in the second half of embryonic brain development and postnatal stages. In postnatal and adult brain, SVCT2 is highly expressed in all choroidal plexus epithelial cells, shown by colocalization with GLUT1 in the basolateral membranes and without MCT1 colocalization, which is expressed in the apical membrane. We confirmed that choroid plexus explant cells (*in vitro*) form a sealed epithelial structure, which polarized basolaterally, endogenous or overexpressed SVCT2. These results are reproduced *in vivo* by injecting hSVCT2wt-EYFP lentivirus into the CSF. Overexpressed SVCT2 incorporates AA (intraperitoneally injected) from the blood to the CSF. Finally, we observed in Guinea pig brain under scorbutic condition, that normal distribution of SVCT2 in choroid plexus may be regulated by peripheral concentrations of vitamin C. Additionally, we observed that SVCT2 polarization also depends on the metabolic stage of the choroid plexus cells.

## Introduction

Choroid plexus epithelial cells are connected by tight junctions that restrict the paracellular movement of molecules between the blood and cerebrospinal fluid (CSF)^1–3^. Thus, the expression of zonula occludens 1 (ZO-1), claudins (1, 2 and 11) and occluding that generate a polarized epithelium (separating the apical membrane that contacts the CSF from the basolateral membrane, which is in contact with the blood) has been demonstrated in these cells^4,5^. Different studies have been carried out to examine choroid plexus function in primary cell cultures and immortalized cell lines^6–10^ using bicameral systems that show different characteristics of polarized epithelial cells^11–15^ and represent a blood-CSF barrier model for transcellular transport studies^13,14,16,17^. However, a limitation of these *in vitro* systems for studying the blood-CSF barrier is the exclusion of key structural components of the choroid plexuses, such as blood capillaries and stromal cells. In this way, choroid plexus explants represent an interesting study model of the blood-CSF barrier. Using this model, the subcellular localization of metal transporters, transferrin receptor (DMT1, MTP1 and TfR), and organic anion transporter (rROAT1-GFP)^18,19^ has been studied. In explants of rat and shark choroidal plexus, transcellular transport and stroma fluorescein accumulation have also been studied^20,21^

Vitamin C is an essential micronutrient for the normal metabolic functioning of the organism^22–28^. It is used as a cofactor in hydroxylation reactions and is a powerful water-soluble antioxidant; its participation in differentiation processes in different cell types has recently been determined^29–39^. In blood plasma, a concentration close to 50 μM has been detected, mainly in its reduced form, ascorbate (AA), with 5–10% in its oxidized form, dehydroascorbic acid (DHA). Independent of the ability to synthesize vitamin C, it must be efficiently incorporated into the different cells of the body. AA is actively incorporated by the cytoplasmic membrane through the sodium-ascorbate cotransporters (SVCTs)^40^ and DHA is transported using the facilitated hexose transporters, GLUTs^41–50^

*In vivo* studies injecting ^14^C-labeled AA and subsequent autoradiography have shown that the radioactivity does not penetrate directly from the blood to the brain^51^. A high concentration of labeled AA was observed in the choroid plexus 2 min after the injection. Radioactivity spread from these areas throughout the brain by 24 h, with a high concentration of AA in the hippocampus and cerebellar cortex^51^. In studies carried out with rabbit choroid plexus cells *in vitro*, it was determined that it incorporated AA through a saturable type transport with an approximate Km of 44 μM^52,53^. Once the sodium-ascorbate cotransporters, SVCTs, were cloned, high SVCT2 mRNA expression was detected in the epithelial cells of the choroid plexus^30,46^. The expression of the mRNA added to the AA incorporation studies was suggestive of the presence of an active, saturable transporter, SVCT2, which would serve to transfer AA from the blood plasma to the CSF and subsequently the brain^54,55^

Using porcine choroid plexus epithelial cells in bicameral culture systems, it was possible to detect active sodium-dependent AA transport in the basolateral membrane^16^. However, attempts to determine the basolateral localization of SVCT2 have failed, and only intracellular expression of SVCT2 has been observed in the epithelial cells of rat choroid plexus^56^. A similar location has been observed in human choroid plexus papilloma cells, even though kinetic studies to analyze AA uptake indicate that SVCT2 is expressed in these cells^57^.

In this work, we have analyzed the polarization of SVCT2 in choroid plexus explants. SVCT2 is widely observed with basolateral polarization in all plexus cells isolated from different ventricles. Its expression is detected early in development, and its polarization can be observed on E15 in the rat brain. In the scorbutic Guinea pig choroid plexus, SVCT2 increases its basolateral polarization, detecting the fall in the plasma vitamin C concentration. Choroid plexus cultured in the absence of glucose showed SVCT2 depolarization; polarization is re-stablished in the presence of glucose or lactate.

## Results

### SVCT2 and GLUT1 are widely expressed in choroid plexus cells

**Figure 1:**
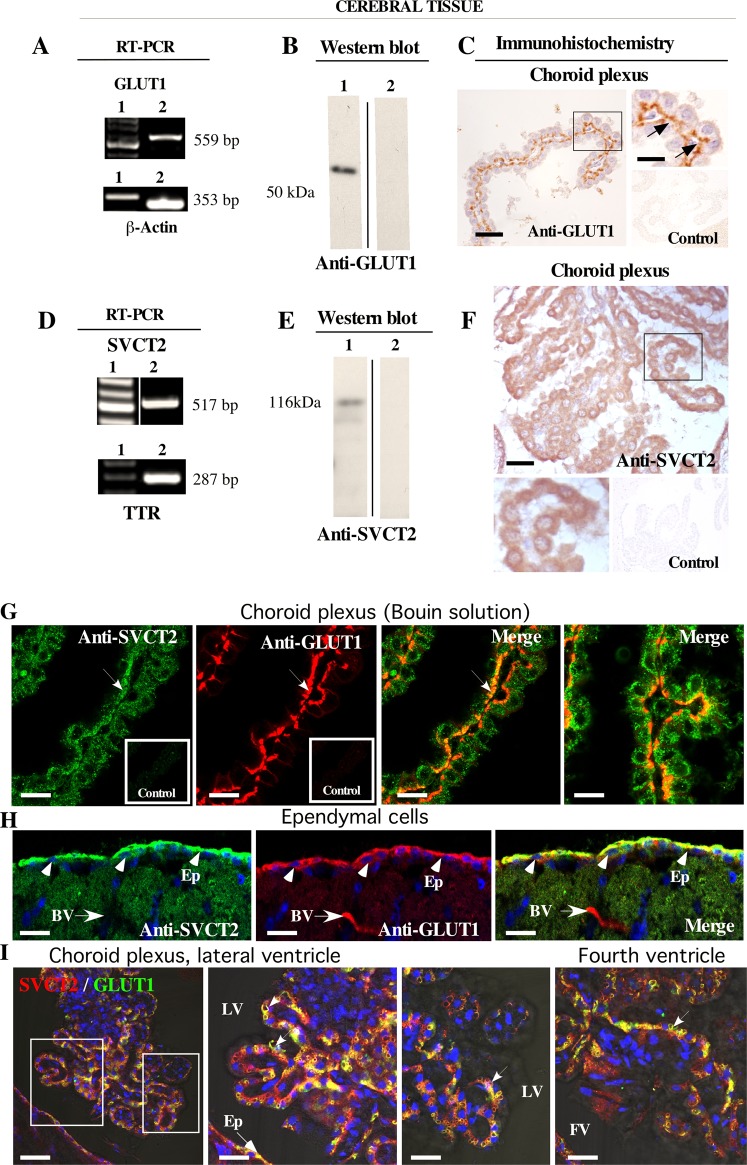
SVCT2 is expressed in choroid plexus cells and only maintains its basolateral polarization in fixed brains with normal physiological levels of glucose. (**A,B,D,E**) RT-PCR and Western blot analysis for GLUT1 and SVCT2 normally expressed in choroid plexus cells. RT-PCR, Line 1. DNA ladders. Line 2. Choroid plexus samples. (**C,F**) Immunohistochemical analysis for GLUT1 and SVCT2 using brains fixed with vascular perfusion of 4% paraformaldehyde without glucose. (**G**,**H**) Immunofluorescence analysis of GLUT1 and SVCT2 using brains fixed with vascular perfusion of Bouin solution without glucose. (**I**) Immunofluorescence analysis of GLUT1 and SVCT2 using brains fixed with vascular perfusion and 4% paraformaldehyde with 5 mM glucose. All images are representative of different biologically independent samples. B, E. Blots cropped from different parts of the same gel. C, F, n = 3. (**G**,**H**), n = 3. I, n = 4. BV. Blood vessel. Ep. Ependymal cells. FV. Fourth ventricle. LV. Lateral ventricle. Scale bars: C and F 30 μm; G–I 15 μm.

We first demonstrated that GLUT1 and SVCT2 are widely expressed in choroid plexus cells from the lateral ventricle of the mouse brain using RT-PCR and Western blot analysis (Fig. 1A,B,D,E). Using sections of brain tissue incubated in 4% paraformaldehyde for 2 days, GLUT1 was detected in the basolateral membrane of choroid plexus cells (Fig. 1C, arrows); however, SVCT2 was mainly internalized (Fig. 1F). Due to this unexpected result, we fixed brain tissues by perfusion with Bouin solution, and immunofluorescence analysis showed that although most SVCT2 is intracellular (Fig. 1G), a fraction was detected at the basolateral membrane, colocalizing with GLUT1 (Fig. 1G, arrow and merge). At the ependymal level of the third ventricle, GLUT1 and SVCT2 were preferentially detected at the apical membrane (Fig. 1H, arrow heads), suggesting differential polarization of SVCT2 in both cell types. Thus, methods of fixation (i.e., perfusion vs immersion) change SVCT2 polarization exclusively in choroid plexus cells, but not in ependymal cells or neurons (data not shown). Because vascular perfusion or immersion techniques induce hypoglycemia, we hypothesize that the polarization of SVCT2 in the choroid plexus is extremely sensitive to glucose levels; therefore, we added glucose to subsequent experiments in which brain tissues were fixed. Under these conditions, SVCT2 and GLUT1 are colocalized in the basolateral membrane of choroid plexus cells (Fig. 1I, arrows). However, the morphology of the epithelial cells of the choroid plexuses was altered, generating vacuoles in the basal region of the cells (Fig. 1I, arrows). Due to this artifact, we isolated the choroid plexus quickly and developed a method of fixation for choroidal plexus explants.

### SVCT2 is mainly polarized to the basolateral membrane in choroid plexus explant cells

**Figure 2:**
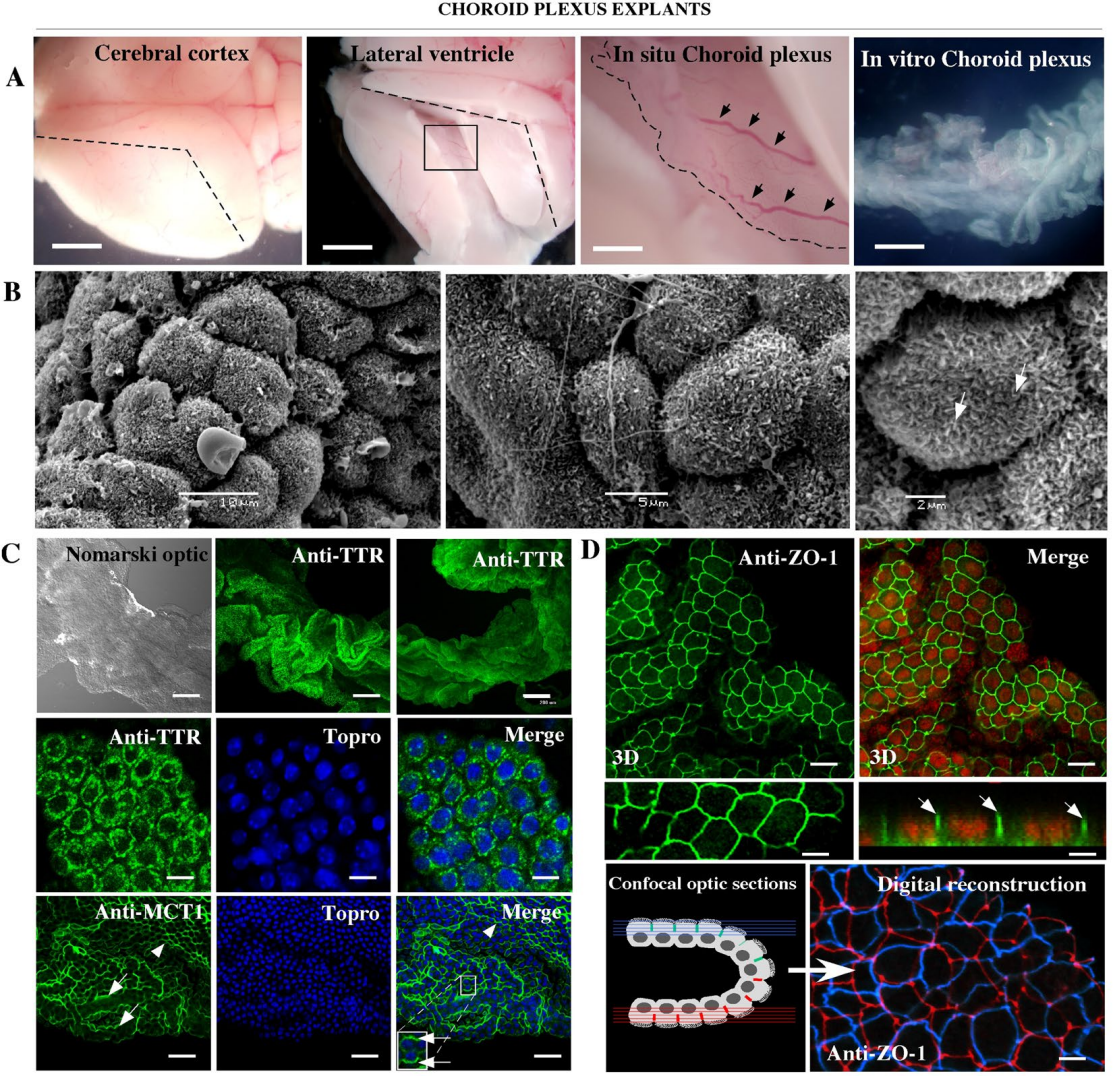
Choroid plexus explants maintain epithelial cell polarization. **(A)** Isolation of choroid plexus from the lateral ventricle. (**B)** Scanning electron microscopy to define normal cell polarization. (**C**) Explant of choroid plexus analyzed using Nomarski optic, or by immunofluorescence and confocal microscopy after anti-TTR or anti-MCT1 incubation. TOPRO-3 was used for nuclear staining. (**D**) Immunofluorescence and confocal microscopy 3D-reconstruction of choroid plexus after anti-ZO1 incubation. Confocal orthogonal reconstruction for ZO-1 identification (arrows). Analysis of ZO-1 distribution after 3D-reconstruction (Imaris software) in the epithelial cell bilayer that forms the choroid plexus. All images are representative of different biologically independent samples. B, n = 3. (**C**,**D**), n = 6. Scale bars: A 1 mm; C 200 μm (lower magnification), 10 μm (higher magnification); D 10 μm (lower magnification), 3 μm (higher magnification).

**Figure 3:**
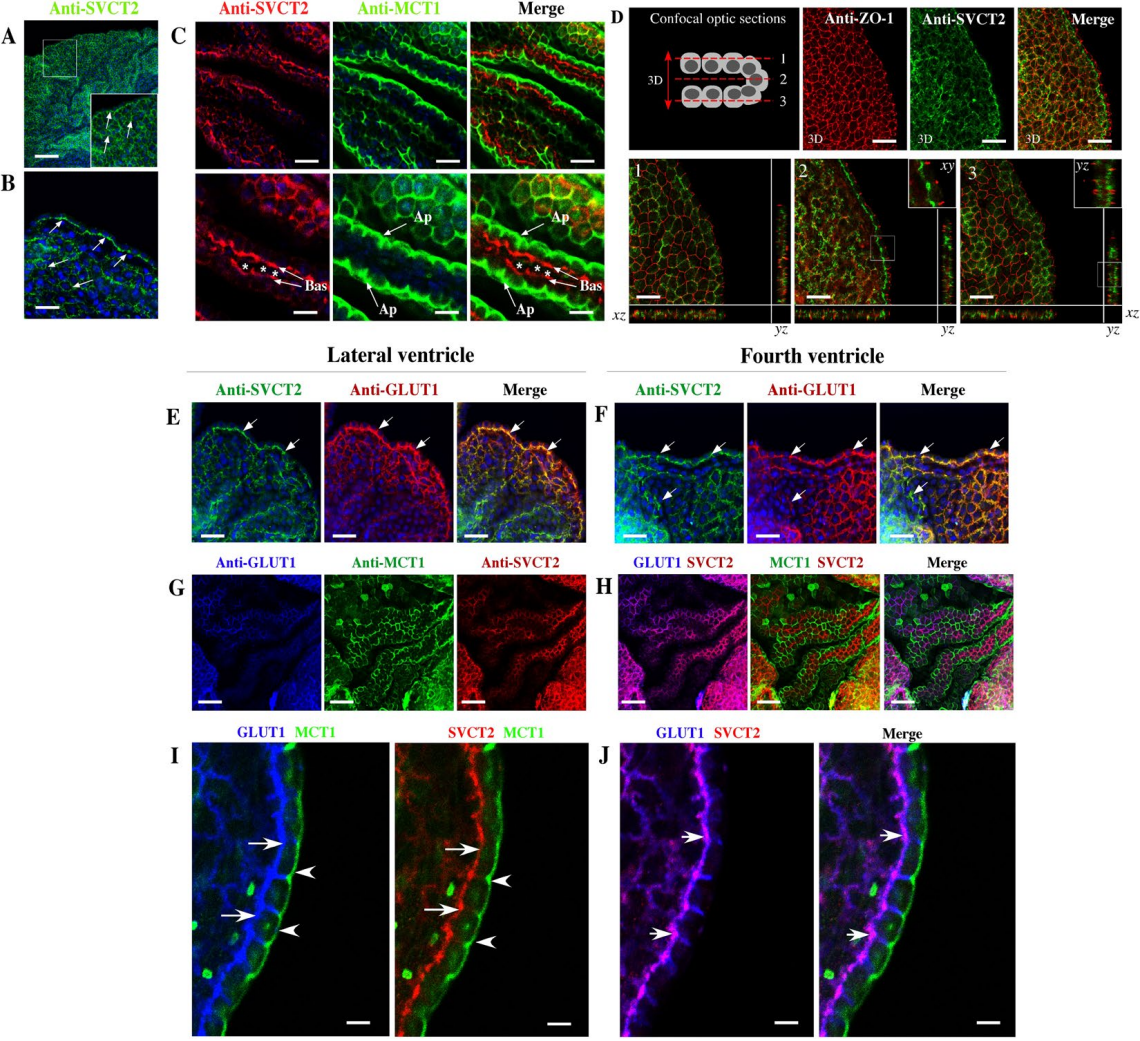
Choroid plexus explants maintain SVCT2 polarization. (**A–C**) Explants of choroid plexus analyzed by immunofluorescence and confocal microscopy after anti-SVCT2 or anti-MCT1 incubation. TOPRO-3 was used for nuclear staining. SVCT2 showed basolateral polarization (Bas), and MCT1 was detected in apical membranes (Ap). (**D**) Immunofluorescence and confocal microscopy 3D-reconstruction of choroid plexus after anti-ZO1 or SVCT2 incubation (3D). Confocal orthogonal microscopy reconstruction for ZO-1 and SVCT2 identification in three different regions inside the epithelial cell bilayer (1,2,3). (**E,G,I**) Immunofluorescence and confocal microscopy 3D-reconstruction of choroid plexus from the lateral and fourth ventricles after incubation with different combinations of antibodies: anti-SVCT2 and anti-GLUT1 (**E**,**F**), anti-GLUT1 and anti-MCT1 (**G**) or anti-GLUT1, anti-MCT1 and anti-SVCT2 (**H**). High-power imaging of confocal spectral microscopy analysis is showed in I and J. GLUT1 and SVCT2 showed the higher colocalization in the basal membrane (J, arrows). All images are representative of different biologically independent samples. A–D, n = 9. E–J, n = 9. Asterisk. Connective tissue. Scale bars: A 100 μm; B, D, G, H 30 μm; C, E, F 20 μm; E–H 50 μm; I–J 8 μm.

Because standard procedures to fix brain tissue do not allow for the detection of SVCT2 basolateral polarization, we standardized the rapid dissection of the choroid plexus (Fig. 2A, arrows) to generate tissue explants maintained for a short time in culture (Fig. 2A, *In vitro* Choroid plexus). The lateral ventricle and fourth ventricle plexus (data not shown) were isolated and maintained as a compact structure in culture (Fig. 2B,C). Scanning electron microscopy showed that the cells remain polarized, forming a continuous epithelium, where the cells present small microvilli on their apical membrane (Fig. 2B, arrows). Using confocal microscopy, we confirmed intracellular transthyretin (TTR) distribution (Fig. 2C) and monocarboxylate transporter 1 (MCT1) apical localization (Fig. 2C, arrows and inset). Finally, ZO-1 was detected at the tight junctions (Figs 2D and 3D reconstruction and orthogonal image, arrows), which maintain the integrity of the epithelial layer (blue and red borders) (Fig. 1D, digital reconstruction). Thus, we conclude that choroid plexus cells *in vitro* maintain the normal polarization of different proteins in their membranes.

SVCT2 was widely detected in the cellular membrane of choroid plexus explant cells (Fig. 3A,B arrows). When performing a comparative analysis with MCT1, we defined that SVCT2 is fundamentally polarized to the basolateral membrane (Fig. 3C, Bas arrows) whereas MCT1 is found at the apical membrane (Fig. 3C, Ap arrows). Using confocal three-dimensional analysis (Fig. 3D, arrow 3D and 3D images), ZO-1 is extensively localized in the apical region of the cells; however, SVCT2 is observed in the basolateral region (Fig. 3D, 3D-images). When analyzing the immunofluorescence in three different optical sections and orthogonal projections (Fig. 3D, images 1, 2 and 3), SVCT2 does not colocalize with ZO-1 (Fig. 3D, images 2 and 3, insets). However, SVCT2 and GLUT1 showed colocalization in the lateral and fourth ventricle choroid plexus cells (Fig. 3E,F Merge-yellow and arrows, and H and Merge-magenta). Finally, we confirmed that MCT1 and SVCT2 are polarized to different cellular membranes (Fig. 3G and H-Merge).

Following triple labeling for SVCT2 (red), MCT1 (green) and GLUT1 (blue) (Fig. 3I,J) (high-power view analysis), GLUT1 is mainly concentrated in the lateral and basal membrane (arrows) with MCT1 in the apical membrane (arrow head) (Fig. 3I). Similarly, SVCT2 is only detected in the basal membrane of the epithelial cells (arrows) with MCT1 in the apical membrane (arrow head) (Fig. 3J). Merge high-power view analysis confirmed GLUT1 and SVCT2 colocalization (arrows-magenta) (Fig. 3J). Thus, differential sorting of transporters is detected in choroid plexus cell explants.

### SVCT2 is expressed in choroid plexus cells during embryonic development with polarized localization in postnatal stages

**Figure 4:**
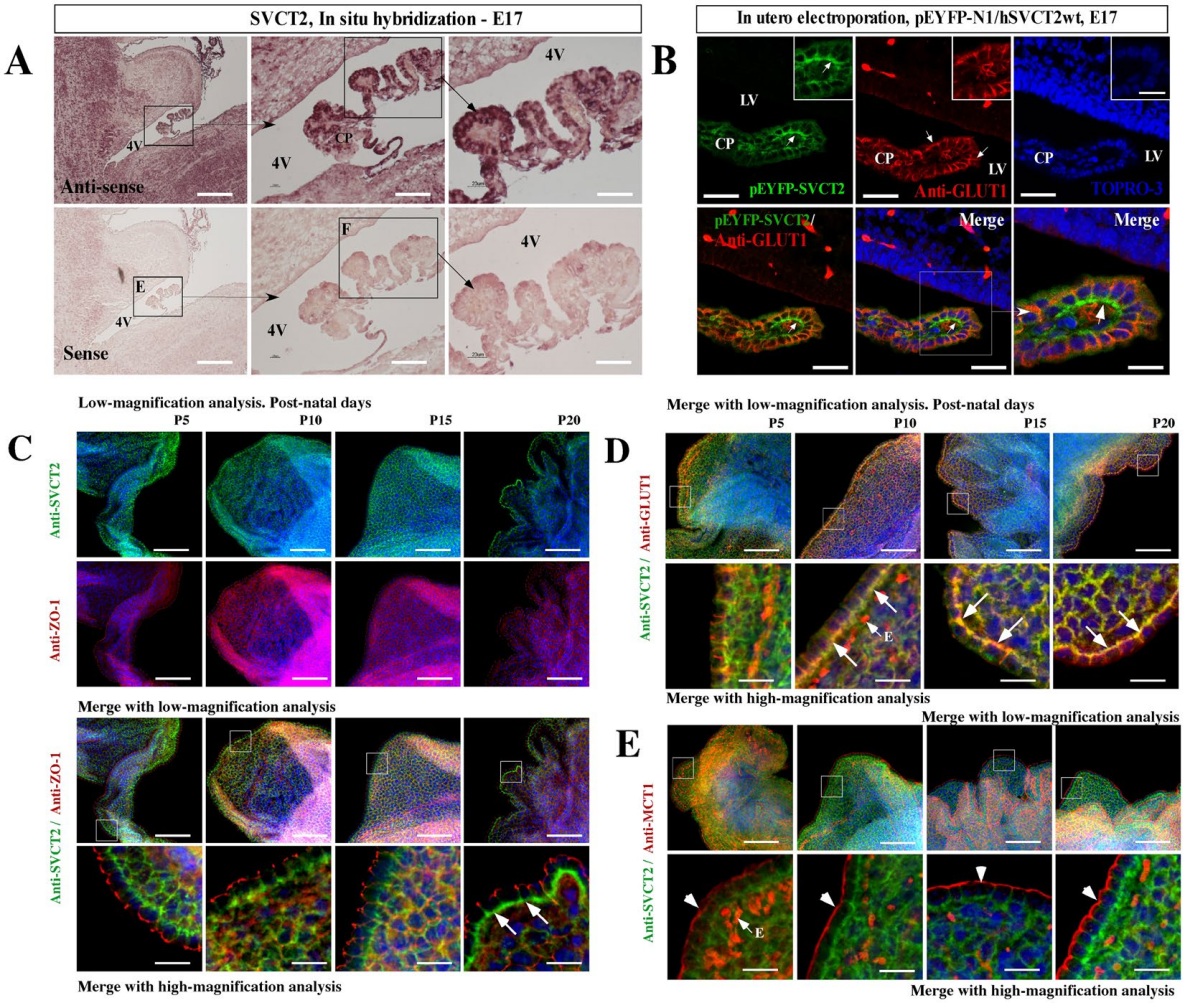
SVCT2 is expressed early during brain development and choroid plexus cell polarization appears after 10 postnatal days. (**A**) *In situ* hybridization analysis of embryonic 17-day-old brains. The choroid plexus of the fourth ventricle had higher SVCT2 mRNA expression. (**B**) *In utero* electroporation analysis using the pEYFP-N1/hSVCT2wt vector in embryonic day 15. Immunofluorescence analysis after anti-GLUT1 incubation; Topro-3 was used for nuclear staining. Merged image indicates that over-expressed SVCT2 is polarized in the basolateral membrane (arrow). (**C**–**E**) Explant of choroid plexus at postnatal days 5, 10, 15 and 20, analyzed by immunofluorescence and confocal microscopy after anti-SVCT2 and anti-ZO1 incubation (**C**), anti-SVCT2 and anti-MCT1 incubation (**E**) and anti-SVCT2 and anti-GLUT1 incubation (**D**). TOPRO-3 was used for nuclear staining. All images are representative of different biologically independent samples. A, n = 3. B, n = 6. C–E, n = 4. 4v. Fourth ventricle. CP. Choroid plexus. E. Endothelial cells. LV. Lateral ventricles. Scale bars: A 100 and 50 μm (lower magnification) and 15 μm (higher magnification); B 20 μm; C–E 50 μm (lower magnification) and 15 μm (higher magnification).

Although SVCT2 mRNA is strongly detected at 17 days of gestation in the rat brain (Fig. 4A), the protein signal is weak with diffuse distribution in cells of the choroid plexus during development (data not shown). To define whether the cells of the choroid plexus have polarized distribution of SVCT2 during brain development, we performed *in utero* electroporation in 15 day-old developing brains as in Silva-Alvarez *et al*. (2016)^58^ to over-express pEYFP-hSVCT2wt and detect its polarization at 17 days. Surprisingly, SVCT2 is clearly polarized in some cells of the plexus that are positively electroporated (Fig. 4B, arrow and inset). By immunofluorescence analysis, GLUT1 is widely expressed in the cellular membranes of the plexus, but without clear polarization (Fig. 4B, arrows). At this stage, SVCT2 and GLUT1 did not colocalize (Fig. 4B, arrows in lower panels and inset).

Because cells of the choroid plexus can polarize SVCT2 during embryonic development, we next defined SVCT2 polarization in postnatal stages P5, P10, P15 and P20 (Fig. 4C,D-E). Using low-magnification analysis, SVCT2 and ZO-1 are widely expressed in these stages (Fig. 4C, upper panels), although without colocalization (Fig. 4C, lower panels, merge with low- and high-magnification analysis and insets). Greater SVCT2 polarization is clearly detected at P20; a thickened positive band is observed in the basal region of the choroid plexus cells (Fig. 4C, arrows). In the same stages, SVCT2 colocalizes with GLUT1 (Fig. 4D, arrows in insets), but not with MCT1 (Fig. 4E, insets and arrow heads). We concluded that SVCT2 is intensely expressed in the second half of embryonic development, showing basolateral polarization that increases strongly during the first 20 days of postnatal development.

### SVCT2 is functionally over-expressed with basolateral polarization in choroid plexus cells using lentiviral vectors *in vitro* and *in vivo*

**Figure 5:**
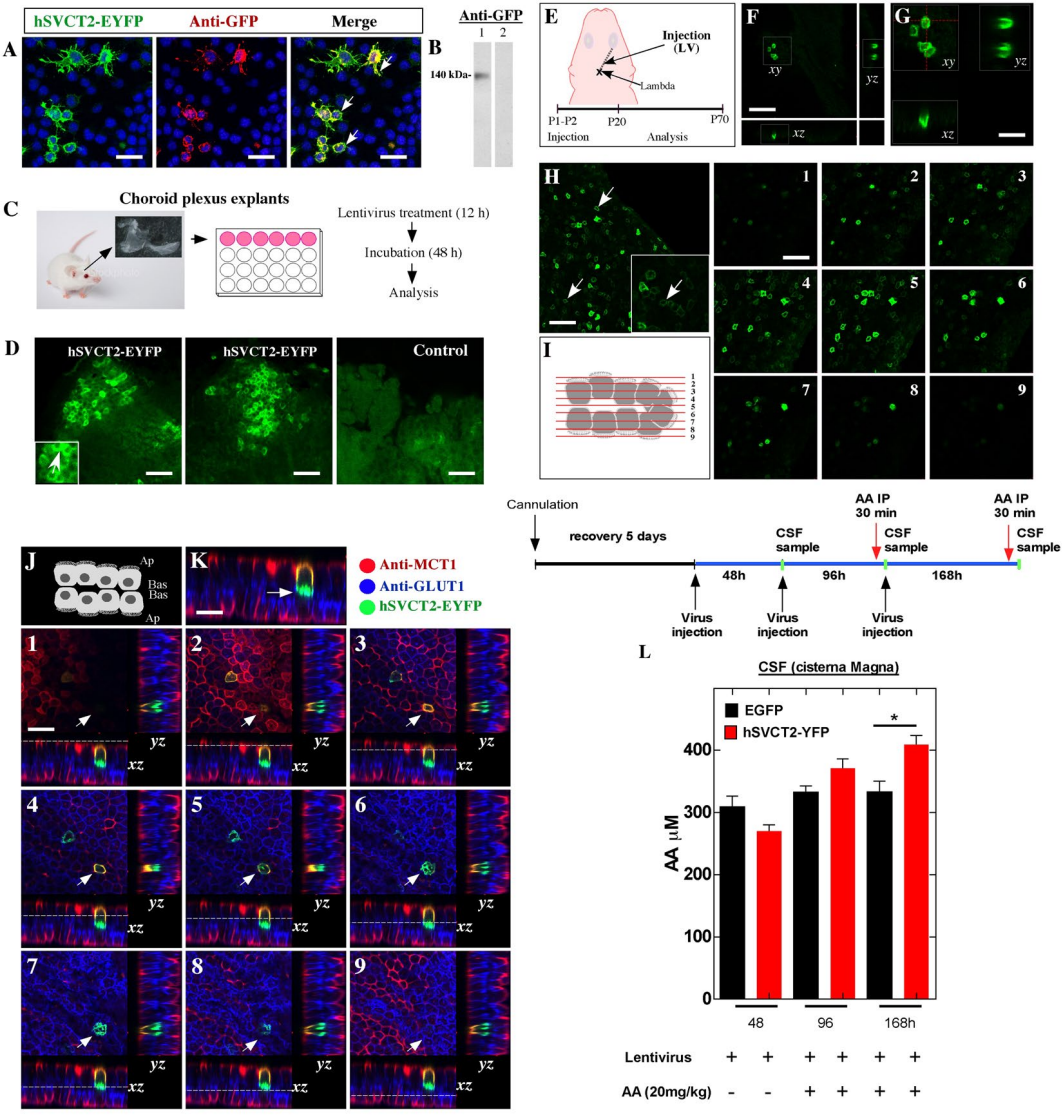
Lentiviral SVCT2 expression in choroid plexus explants *in vitro* and *in vivo*. **(A)** hSVCT2-EYFP lentivirus overexpression and anti-GFP detection in Neuro2a cells. TOPRO-3 was used for nuclear staining. (**B**) Western blot analysis of samples isolated from Neuro2a cells (line 1) with hSVCT2-EYFP overexpression and incubated with anti-GFP. Line 2. Control. (**C,D**) Choroid plexus explants with lentiviral treatments and immunofluorescence microscopy analysis. (**E**) Lentivirus overexpressing hSVCT2-EYFP was injected at P1-P2 and was analyzed at P20 or P70. (**F,G**) Confocal microscopy analysis and orthogonal projection of choroid plexus cells after 20 days of lentiviral lateral ventricle injection. (**H**,**I**) Confocal microscopy analysis of Z-stack analysis (I) after 70 days of lentiviral lateral ventricle injection. The different optical sections are shown in images 1–9. (**J,K**) Confocal microscopy analysis of choroid plexus after 20 days following injection of a lentivirus over-expressing hSVCT2-EYFP and incubated with anti-GLUT1 and anti-MCT1. Confocal z-stack imaging and orthogonal projections (*xz* and *yz*) in different optical sections (1–9). Arrow, positive cell for SVCT2, GLUT1 and MCT1. (**L**) Increased transfer of vitamin C inside the CSF after lentiviral over-expression of the hSVCT2-EYFP transporter *in vivo*. Three different samples of CSF from the cisterna magna were analyzed after lentivirus injections (48, 96 and 168 h). Thirty min before the last two CSF samples were harvested, the animals were injected with intra-peritoneal AA. All images are representative of different biologically independent samples. A, n = 3. D, n = 3. F–H, n = 4. K, n = 3. L, data are shown *t*-student (two-tailed) as mean ± SEM; all data are representative of three separate experiments. * *p* ≤ 0.05. Scale bars: A, K 20 μm; D 40 μm, F–G 20 μm; H 30 μm.

Using a lentivirus that over-expresses hSVCT2wt-EYFP, which efficiently transduces Neuro2a cells (Fig. 5A), SVCT2 is polarized in the plasma membrane (Fig. 5A, arrows); a positive reaction for anti-GFP is also shown. Epithelial cells of the choroid plexus explants are also transduced *in vitro* by hSVCT2wt-EYFP lentivirus (Fig. 5C,D). Thus, hSVCT2wt-EYFP lentivirus was injected into the lateral ventricle at 1 day to analyze the expression and polarization of SVCT2 at days 20 and 70 (Fig. 5E). A significant number of cells were positive for hSVCT2wt-EYFP at days 20 (Fig. 5F,G) and 70 (Fig. 5H,I), where SVCT2 was detected in the cell membrane of the transduced epithelial cells (H, arrows). 3D-reconstruction of the positive cells (Fig. 5G, *xy*) shows SVCT2 in the silhouette of the cells, which is confirmed in the orthogonal planes *xz* and *yz*, showing SVCT2 basolateral polarization (Fig. 5G). Using 3D-reconstruction, we analyzed positive cells in 9 optical confocal planes (planes 1–9), confirming that SVCT2 is found in the cellular membranes of transduced cells (Fig. 5I, 1–9 images). Additionally, SVCT2-overexpressing cells also express GLUT1 in the basolateral membrane and MCT1 in the apical membrane (Fig. 5J,K). The highest SVCT2 colocalization is observed with GLUT1 (Fig. 5K, arrow), which is confirmed in 9 optical planes with *yz* and *xz* orthogonal projection (Fig. 5K, optical planes 1–9 with positive cell-arrow).

Finally, using a fixed cannula with a stereotaxic frame in the lateral ventricle, three injections of hSVCT2wt-EYFP lentivirus (see experimental scheme) were made in the lateral ventricle, and CSF samples were obtained from the cisterna magna at 48, 96 and 168 h. Thirty minutes before the second and third CSF extraction (96 and 168 h), AA was injected intraperitoneally for 30 min, and its concentration was analyzed in the CSF using the FRACS method. We did not observe significant differences in the concentration of AA in the CSF at 48 or 96 h of control animals or those intraperitoneally injected with AA. However, at 168 h after the first injection of the hSVCT2wt-EYFP lentivirus, a significant increase in AA transfers to the CSF in animals overexpressing SVCT2 (Fig. 5L). Thus, SVCT2, which can be over-expressed functionally in choroid plexus cells *in vivo*, is localized to the basolateral membrane by incorporating AA from the blood to the CSF.

### The choroid plexus explants from scorbutic Guinea pigs increase SVCT2 basolateral polarization

**Figure 6:**
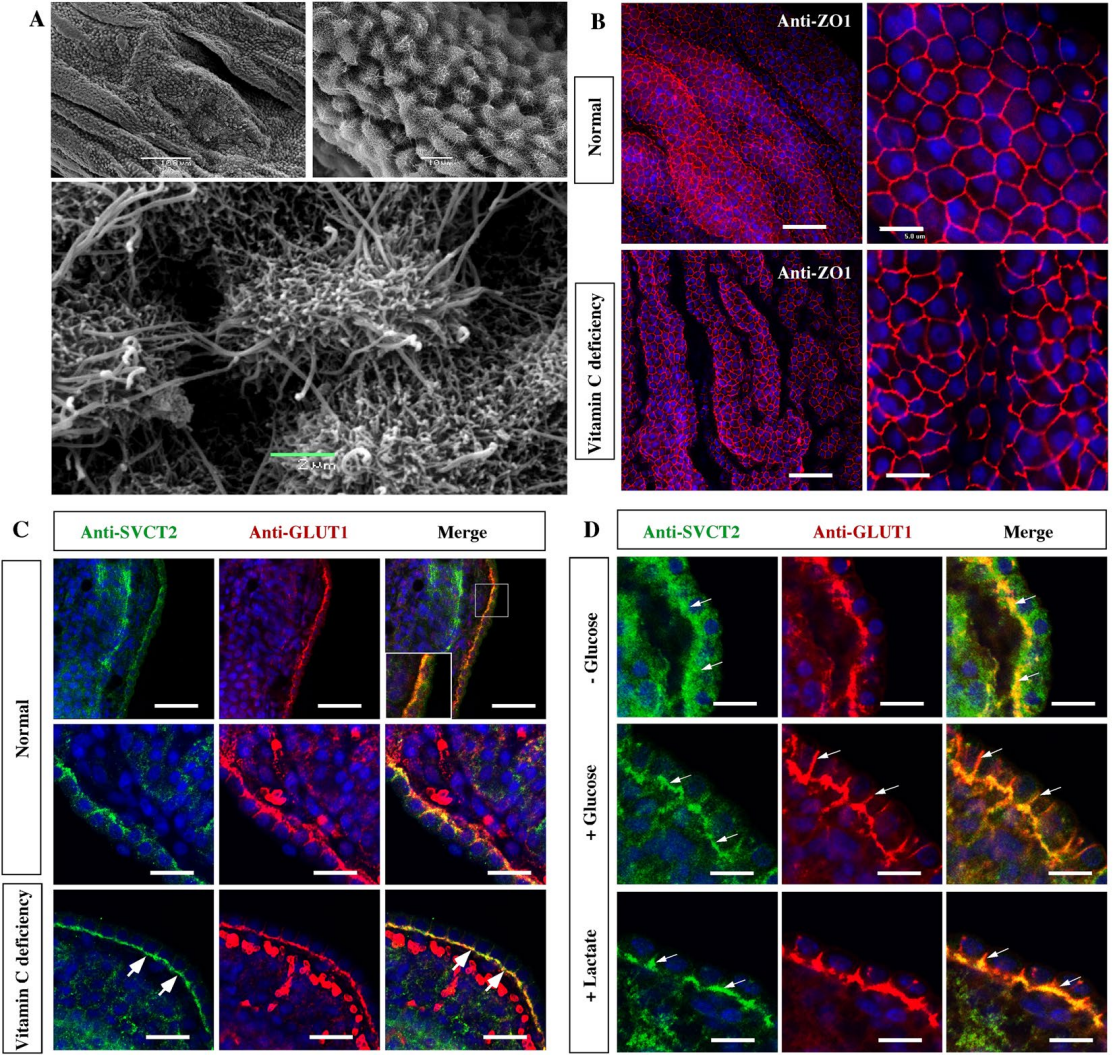
Vitamin C deficiency and normal energetic culture demands increase SVCT2 basolateral polarization in choroid plexus cells from Guinea pig. (**A**) Scanning electron microscopy of choroid plexus explants after induction of vitamin C deficiency. (**B**) Confocal microscopy analysis of choroid plexus explants maintained in normal or vitamin C deficiency conditions after incubation with anti-ZO. TOPRO-3 was used for nuclear staining. (**C**) Confocal microscopy analysis of choroid plexus cells after anti-SVCT2 and anti-GLUT1 incubation in normal or vitamin C deficiency conditions. (**D**) Glucose and lactate induce SVCT2 re-polarization in the basolateral membrane of choroid plexus cells cultured without glucose (7 h). All images are representative of different biologically independent samples. A, n = 2. B, n = 2. C–D, n = 6. Scale bars: B 30 μm (lower magnification) and 5 μm (higher magnification); C 50 μm (lower magnification) and 20 μm (higher magnification); D 20 μm.

We induced vitamin C deficiency in Guinea pig brains to define whether the polarization of SVCT2 can be modulated *in vivo* by the scorbutic condition. First, we defined that the choroid plexus of scorbutic animals maintains the barrier structure (Fig. 6A) at the ultrastructural level, in addition to its polarization. Microvilli and cilia were observed on the apical side of the cells. We also detected that ZO-1 expression was maintained in the explant cells generating a bee-like panel image (Fig. 6B). Under normal feeding conditions with vitamin C in the diet, SVCT2 is polarized to the basolateral membrane, colocalizing with GLUT1 (Fig. 6C and inset). An intense reaction for GLUT1 is also observed in red blood cells. However, in the epithelial cells of the Guinea pig plexus, the reaction is heterogeneous in different cells (Fig. 6C, high-magnification images). Under vitamin C deficiency, SVCT2 is observed with great intensity, forming a well-defined, continuous band in the basal region of the cells (Fig. 6C, arrows), showing colocalization with GLUT1 (Fig. 6C, arrows). These results suggest that vitamin C deficiency increases SVCT2 polarization in the choroid plexus; thus, SVCT2 expression can be regulated by *in vivo* vitamin C concentration.

Finally, we used choroid plexus explants to define whether glucose deprivation changes SVCT2 polarization (Fig. 6D). Under these conditions, SVCT2 loses its polarization rapidly (Fig. 6D, arrows), a condition that is recovered by adding glucose as well as lactate to the medium (Fig. 6D, +Glucose or +Lactate, arrows). Interestingly, the absence of glucose does not affect GLUT1 polarization. These data suggest that the normal distribution of SVCT2 in the choroid plexus depends on the metabolic stage of the cells, which is heavily altered during brain tissue immersion or perfusion.

## Discussion

Vitamin C enters the brain continuously through the choroid plexus, epithelial cells that form the blood-CSF barrier. Although the transfer of AA to the brain through the choroid plexus has been measured *in vivo*^52,53^, SVCT2 polarization in cells of the choroid plexus has not been defined histologically. Unexpectedly, we observed that the polarization of SVCT2 is sensitive to glucose levels in the vascular perfusion fluid. Using fixatives without glucose, SVCT2 is internalized and within cytoplasmic compartment. However, when glucose is incorporated into the fixer fluid, virtually 100% of SVCT2 has basolateral distribution and colocalizes with GLUT1. Unfortunately, a fixative solution with glucose alters the tissue structure, generating cells with osmotically altered membranes with a “bubble’’ appearance. To overcome this experimental difficulty, we prepared choroid plexus explants performing accelerated brain dissection, which after a few moments in culture medium, were fixed. Under these conditions, excellent tissue preservation was maintained with perfect ZO-1 distribution, as well as MCT1 apical localization and GLUT1 basolateral sorting. In addition, the TTR transporter was preferably observed intracellularly. We conclude that the choroid plexus explants maintain a cellular and functional structure similar to that observed *in vivo*.

Epithelial cells of the choroid plexus explants maintain interaction with the extracellular matrix, which regulates cellular polarization and maintains the histological structure of the barrier. Thus, this organoid-like structure should better maintain the barrier properties of the choroid plexus cells compared to cell cultures in monolayers using Transwell systems, which have been previously used to analyze transcellular vitamin C transport *in vitro*^11,16^. We showed that epithelial tight junctions were perfectly formed and maintained. In these conditions, we precisely defined the basolateral localization of SVCT2 in most choroid plexus cells. Our SVCT2 detection method allows us to conclude that the transporter is massively expressed in most of the epithelial cells of the choroid plexus. SVCT2 has a basolateral distribution in the cells, colocalizing with GLUT1. SVCT2 detection at the intracellular level was weak, and colocalization with TTR was not observed. Colocalization with MCT1, an apical transporter, was also not observed.

The phenotype of a polarized epithelium and the large amount of mitochondria present in the epithelial cells of the choroidal plexuses are characteristic of secretory epithelia^59^, which need high energy to maintain cellular processes related to transport and secretion. Acute hypoglycemic conditions lead to SVCT2 internalization and probably cause transient vitamin C deficit within the CSF. Long periods of starvation or malnutrition may directly affect SVCT2 sorting, decreasing the entry of vitamin C into the brain and may increase oxidative damage. These dietary conditions may also affect SVCT2 sorting in other tissues, probably decreasing the level of vitamin C in many peripheral organs; however, this condition must be studied. SVCT2 seems to have different types of sorting in different cells, which may each be affected by the hypoglycemic condition. For example, SVCT2 is located in the apical membrane in ependymal cells (present work), in rat epithelial cells of the bronchiole, and in syncytiotrophoblasts of the human placenta^60^. Following over-expression, basolateral SVCT2 has been observed in renal and intestinal epithelial cell lines^61,62^, as well as epithelial cells of the proximal and distal tubules of the kidney during postnatal development^63^. The basolateral polarization of hSVCT2 over-expressed in MDCK and Caco-2 cells^62,64^ depends on a basolateral amino acid sequence (LMAI) present in the amino terminal domain, between amino acids 56 and 59. Although some domains that regulate its sorting are known, the pathophysiological conditions can regulate SCVT2 basolateral polarization are unknown. In this study, we have defined that hypoglycemia may modify the sorting of SVCT2. Additionally, we have analyzed other two conditions, i) early postnatal brain development and ii) vitamin C deficiency.

Formation of the choroid plexus occurs early in development^2,65–68^. By i*n situ* hybridization, we confirmed SVCT2 mRNA expression in the rat choroidal plexus at 11 days of brain development^58^, however, immunohistochemical detection without polarization was observed at 15 days of development^58^. By *in utero* electroporation, we observed the polarization of hSVCT2-EYFP overexpressed in the choroidal plexus of 17-day-old mice, prior to the polarization of GLUT1. Therefore, SVCT2 is polarized early during brain development, probably during the blood-CSF barrier formation, and its location does not vary during postnatal development. GLUT1 polarization is induced postnatally, and is likely associated with the closure of the blood-brain barrier (BBB)^69^, condition that does not seem to regulate SVCT2 polarization. In this period, the CNS preferentially uses ketone bodies to obtain energy. In addition, there is an increase in the expression of monocarboxylate transporters, such as MCT1^70^. GLUT1 polarization in the cells of the choroid plexus and in the endothelial cells of the BBB coincides with dietary changes, where the brain begins to use glucose to supply the energy requirements. However, the data obtained for SVCT2 in the choroid plexus indicate that this transporter acquires its polarization early in embryonic development, and it remains polarized during postnatal life, regulating the concentrations of brain vitamin C independent of BBB postnatal formation^71,72^.

Vitamin C deficiency *in vivo* could be a determining factor in the basolateral polarization of SVCT2. Most mammals synthesize AA from glucose. However, species, such as human and Guinea pigs, must incorporate vitamin C into their diet as they lack L-gulono-γ-lactone oxidase, which is necessary for AA biosynthesis^73^. Generation of vitamin C deficiency in Guinea pigs by reducing their intake for 4 weeks produced greater SVCT2 polarization in epithelial cells of the choroidal plexus, probably as a way to optimize incorporation of the circulating vitamin C in these animals. We also analyzed if the metabolic condition affected SVCT2 and GLUT1 polarization. Choroidal plexus express MCT1 and MCT3, in addition to GLUT1, suggesting that these cells can capture lactate and glucose for energy. In the absence of these energy substrates, we observed a deregulation of the basolateral polarization of SVCT2 but not GLUT1, which also was detected in the basolateral membrane. This effect was reversed with both glucose and lactate. Modification of the localization of SVCT2 and GLUT1 suggests that the secretory cells need energy to maintain transport processes and polarization of the transporters.

Our laboratory has been the first to use lentiviral vectors to overexpress SVCT2 transporter *in vivo* and *in vitro* in the choroid plexus. We analyzed the overexpression of hSVCT2-EYFP in the choroid plexus *in vivo* using intracerebroventricular injections. The fluorescence pattern of hSVCT2-EYFP in most cells *in vivo* was similar to the immunofluorescence pattern observed in explants. Overexpression of the transporter in the choroid plexuses *in vivo* demonstrated the basolateral polarization of SVCT2 in a model of endogenous polarization of non-transformed cells. *In vivo* SVCT2 overexpression also allowed us to increase the concentration of vitamin C in the CSF. In this way, similar experimental tools can be designed in the future to compensate for decreased vitamin C in the CSF during aging or during a neurodegenerative disease.

We concluded that SVCT2 is intensely expressed in the second half of embryonic brain development, showing basolateral polarization that increases strongly during the first 20 days of postnatal development. In adult choroid plexus cells, SVCT2 showed high colocalization with GLUT1 in the basolateral membranes of epithelial cells. Additionally, we confirmed that epithelial cells of the choroid plexus *in vitro* and *in vivo* are transduced by an hSVCT2wt-EYFP lentivirus, where SVCT2 was detected in the basolateral membrane of the epithelial cells, which incorporate AA (intraperitoneally injected) from the blood to the CSF *in vivo*. Finally, we induced vitamin C deficiency in Guinea pig brain to define whether the polarization of SVCT2 can be modulated by the scorbutic condition. These data suggest that the normal distribution of SVCT2 in the choroid plexus depends on the metabolic stage of the cells, which is heavily altered during brain tissue immersion or perfusion.

Increasing intracerebral concentrations of vitamin C is a complex process; however, by modifying the expression of SVCT2 in the choroid plexus, it is feasible to increase the concentrations of vitamin C in the CSF, a condition that could help in brain pathologies associated with aging and oxidative damage.

## Materials and Methods

### Animals

Animal work, procedures and methods were approved by the Ethics and Animal Care and Use Committee of the University of Concepcion (Chile), Grant Fondecyt 1181243, therefore, all methods were performed in accordance with the relevant guidelines and regulations of the same University. C57BL/J6 mice (pregnant females, postnatal and adult females and males) were obtained from the University of Concepcion, Department of Cellular Biology Breeding Center. The Guinea pigs were acquired at the Institute of Public Health (ISP), Santiago, Chile. Pirbright Guinea pigs were fed pellets for rabbits with fresh vegetables. Pirbright Guinea pigs with vitamin C deficiency were only fed rabbit pellets for 3 weeks. The animals were maintained on a 12-h light/12-h dark cycle with food and water *ad libitum*.

### Immunohistochemistry and confocal microscopy

Choroid plexus samples from adult rats were dissected and fixed by immersion in 4% paraformaldehyde (PFA) or cultured in DMEM for 3 h. The endogenous peroxidase activity of xylene-deparaffinized, rehydrated sections (7 μm) was inhibited by treatment with 0.3% H_2_O_2_ in methanol. For immunohistochemical analysis, the antibodies were diluted in Tris-HCl phosphate buffer (10 mM Tris, 120 mM NaCl, 8.4 mM Na_2_HPO_4_, 3.5 mM KH_2_PO_4_, pH 7.8) containing 1% bovine serum albumin (BSA) and 0.2% Triton X-100^74^. We used anti-SVCT2 or anti-GLUT1 antibodies, diluted 1:100, and incubated with the tissue sections or choroid plexus explants overnight at 22 °C. After extensive washing, the sections were incubated for 2 h at 22 °C with peroxidase-labeled anti-rabbit IgG (1:500; Jackson ImmunoResearch, West Grove, PA, USA). The peroxidase activity was developed using diaminobenzidine and H_2_O_2_. As a negative control, we omitted the primary antibody. Triple and quadruple labeling for confocal microscopy was performed by co-incubating the sections overnight at 22 °C with anti-SVCT2 (1:100, Santa Cruz Biotechnology, Santa Cruz, CA, USA) and the following antibodies: anti-MCT1 (1:200; Promega, Madison, WI, USA), anti-GLUT1 (1:50; Millipore, Temecula, CA, USA), anti-ZO-1 (1:200, Chemicon International, Inc, Temecula, CA, USA), anti-TTR (1.100, Dako, Carpinteria, CA, USA). After extensive washing, the sections were incubated for 2 h with Cy^2^-conjugated affinity-purified donkey anti-rabbit IgG (1:200; Jackson ImmunoResearch), Cy^3^-conjugated affinity-purified donkey anti-mouse IgG (1:200; Jackson ImmunoResearch), Cy^5^-conjugated affinity-purified donkey anti-chicken IgG (1:200; Jackson ImmunoResearch), and TOPRO-3 (1:500, Molecular Probes, Eugene, Oregon, USA) for nuclear staining.

### *In situ* hybridization

Analysis of SVCT2 mRNA in fetal rat brain was performed as previously described^58^. Briefly, a cDNA of 0.62 kb encoding the rat SVCT2 sequence obtained by RT-PCR from cultured embryonic neurons was subcloned into pCR-4-Blunt-Topo (Clontech, Palo Alto, CA, USA) and used to generate sense and antisense digoxigenin (DIG)-labeled riboprobes by *in vitro* transcription with T3 or T7 RNA polymerase (Boehringer Mannheim, Mannheim, Germany). *In situ* hybridization was performed on fetal brain sections mounted on poly-L-lysine-coated glass slides. The sections were deparaffinized, rehydrated and treated with proteinase K (1 mg/mL in PBS; 3 min at 37 °C). Tissue samples were then fixed with 4% PFA, acetylated for 10 min at 20 °C, incubated in pre-hybridization solution for 15 min at 37 °C and 25 mL of hybridization mix with 1:20 to 1:100 dilutions of riboprobe at 42 °C overnight^40^. Samples were rinsed in SSC (0.15 M NaCl/0.015 M sodium citrate) and washed twice for 15 min at 42 °C. Finally, the slides were washed at 37 °C for 15 min each in 2x SSC, 1x SCC, 0.3x, and 0.03x SSC. Visualization of DIG was performed with a monoclonal antibody coupled to alkaline phosphatase (1:500, anti-DIG-alkaline phosphatase Fab fragments; Boehringer Mannheim) for 2 h at 20 °C. NBT/BCIP (Boehringer Mannheim) was used for colorimetric detection of alkaline phosphatase activity. Controls included use of the sense riboprobe and omission of the probe as previously specified^75,76^.

### RT-PCR analysis

For RT-PCR analysis, we used samples from choroid plexus explants. Total RNA was purified using Trizol reagent (Invitrogen, Carlsbad, CA, USA) and quantified in a SmartSpec300 spectrophotometer (Bio-Rad, Hercules, CA, USA). For RT-PCR, 1 μg of RNA was pre-treated with DNase I (Fermentas, ON, Canada) and processed. The basic thermocycling conditions included the following: one cycle at 95 °C for 5 min; 35 cycles at 95 °C for 30 s, 60 °C for 30 s, and 72 °C for 30 s; and one cycle at 72 °C for 7 min. The following primers were used to analyze the expression of SVCT2: forward 5′-ACGTTTGGATGCAGGTTACCC-3′ and reverse 5′-TGAAGCAGAGCAGCCAGGATAC-3′ [expected product 517 base pairs (bp)] and GLUT1: forward 5′-CATGTATGTGGGGGAGGTGT-3′ and reverse 5′-GACGAACAGCGACACCACAG-3′ [expected product 559 base pairs (bp)].

### Immunoblotting

Samples from choroid plexus explants were obtained by ultrasonic homogenization in ice-cold isotonic buffer containing protease inhibitors as described^40^. Total protein fractions were collected from the supernatant after sedimentation of debris and nuclei (10,000 × g for 10 min at 4 °C). Fifty micrograms of protein were loaded in each lane, separated by sodium dodecyl sulfate–polyacrylamide gel electrophoresis, transferred to PVDF membranes, and probed overnight with anti-SVCT2 (1:200; Santa Cruz Biotechnology), anti-GLUT1 (1:200) or anti-βactin (1:1000; Santa Cruz Biotechnology) antibodies. Secondary antibodies used were conjugated to HRP (1:5000; Jackson Immuno Research), the reaction was developed using the ImageQuant LAS500 enhanced chemiluminescence system (General Electric, Fairfield, Connecticut, USA).

### *In utero* electroporation

Plasmids were prepared according to the manufacturer’s protocol (HiSpeed plasmid midi Kit, Qiagen). After pregnant, rats were anesthetized with isoflurane (2–4%), a midline abdominal incision was performed, and the uterus was taken out. Microinjections of 1–3  uLof plasmid DNA solution (0.5–1 mg/mL) in PBS plus Fast Green (0.05% w/v) into the lateral cerebral ventricles of E14-E15 embryos were made through the uterine wall via a Hamilton syringe. Between 0.5 and 1 µg of EYFP-N1 or EYFP-N1-SCT2wt (n = 5) were used per brain embryo. After injection of the plasmids, five pulses of 50 V each were delivered across the head of the embryo within the uterus^68^. Finally, embryos were reincorporated into the abdominal cavity and allowed to develop for an additional 2–3 days. Electroporated animals were then sacrificed; the brains were fixed, and thick sagittal sections (40–60 mm) were obtained. The position and morphology of transfected cells were analyzed by fluorescent microscopy.

### AA measurement

The animals were anesthetized with 5% isoflurane, and kept at 2% during the procedure. The temperature was kept under control, and the heart rate was monitored in real time using the small animal physiological monitoring system (Hardvard apparatus # 75–1500). In total, 30 µL of CSF were extracted from the cisterna magna by stereotaxic technique, and 20 µL was used to determine AA levels by FRASC colorimetric assay (bioassay system # EASC-100) according to the manufacturer’s instructions.

### Statistical analysis

The statistical analysis was performed using Prism version 4.0 software (GraphPad Software, San Diego, CA, USA). The data represent the mean ± SD of three independent experiments with each determination, which were conducted in triplicate. Statistical comparison between two or more groups of data was carried out using analysis of *t*-student (two-tailed). p < 0.001 was considered statistically significant.

## Supplementary information

Supplementary Info 1

## Data Availability

All data generated or analysed during this study are included in this published article (and its Supplementary Information files).
