# Supplementary material for: Basal Sodium-Dependent Vitamin C Transporter 2 polarization in choroid plexus explant cells in normal or scorbutic conditions
Source: Sci Rep. 2019 Oct 8;9:14422. doi: 10.1038/s41598-019-50772-2 (PMC6783570; doi:10.1038/s41598-019-50772-2)
Supplement: Supplementary file 1 — Supplementary Info 1 [file 41598_2019_50772_MOESM1_ESM.pdf]

Supplementary Information.

Basal Sodium-Dependent Vitamin C Transporter 2 polarization in choroid plexus explant cells in normal or scorbutic conditions

Viviana Ulloa[1], Natalia Saldivia[1], Luciano Ferrada[1], Katterine Salazar[1,2], Fernando Martínez[1,2], Carmen Silva-Alvarez[1], Rocio Magdalena[1], María José Oviedo[1], Hernán Montecinos[3], Pablo Torres[1], Manuel Cifuentes[4], Francisco Nualart[1,2,*]

*Correspondence. F. Nualart, Departamento de Biología Celular, Facultad de Ciencias Biológicas, Universidad de Concepción, Casilla 160-C, Concepción, Chile; E-mail: frnualart@udec.cl

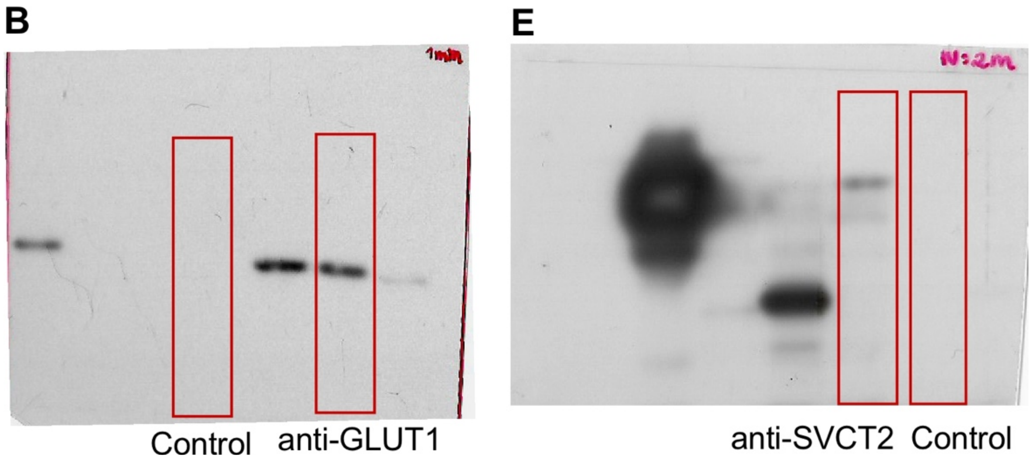

Fig 1. Uncropped blots related to Figure 1B, E.
